# Seasonal forecasts offer economic benefit for hydrological decision making in semi-arid regions

**DOI:** 10.1038/s41598-021-89564-y

**Published:** 2021-05-19

**Authors:** Tanja C. Portele, Christof Lorenz, Berhon Dibrani, Patrick Laux, Jan Bliefernicht, Harald Kunstmann

**Affiliations:** 1 Karlsruhe Institute of Technology (KIT), Campus Alpin, Institute of Meteorology and Climate Research - Atmospheric Environmental Research (IMK-IFU), Garmisch-Partenkirchen, Germany; 2 Tractebel Engineering GmbH, Bad Vilbel, Germany; 3 University of Augsburg, Institute of Geography, Augsburg, Germany

**Keywords:** Hydrology, Hydrology, Climate-change mitigation, Sustainability, Climate sciences, Climate-change mitigation

## Abstract

Increasing frequencies of droughts require proactive preparedness, particularly in semi-arid regions. As forecasting of such hydrometeorological extremes several months ahead allows for necessary climate proofing, we assess the potential economic value of the seasonal forecasting system SEAS5 for decision making in water management. For seven drought-prone regions analyzed in America, Africa, and Asia, the relative frequency of drought months significantly increased from 10 to 30% between 1981 and 2018. We demonstrate that seasonal forecast-based action for droughts achieves potential economic savings up to 70% of those from optimal early action. For very warm months and droughts, savings of at least 20% occur even for forecast horizons of several months. Our in-depth analysis for the Upper-Atbara dam in Sudan reveals avoidable losses of 16 Mio US$ in one example year for early-action based drought reservoir operation. These findings stress the advantage and necessity of considering seasonal forecasts in hydrological decision making.

## Introduction

Climate extremes such as droughts and anomalous wet conditions greatly affect economic wealth, particularly in climate sensitive semi-arid regions^1^. 40% of the global land area are drylands, inhabited by more than one third of the global population^2^ with population growth rates up to 4% per year^3^. For the majority of drylands, especially for semi-arid regions, sustainable water management can be key with respect to the food-energy-water nexus. More than one third of semi-arid drylands are cultivated rain-fed or irrigated farmland^2^. The importance of hydropower generation for states comprising semi-arid regions can be immense with up to two thirds of a state’s total power generated at hydroelectric dams^4^. With increasing frequencies and intensities of droughts and hot extremes, as well as with increasing precipitation variabilities^1,5–8^, conflicts of water use and impacts of climate change may become critical, increasing the importance of sustainable water management and climate proofing in semi-arid regions.

This study addresses the crucial question of whether proactive drought- and extreme event preparedness as well as risk mitigation in water management could be provided by the use of seasonal climate forecasts. Decision makers still hesitate to use seasonal forecasts^9,10^. They claim a lack of confidence and credibility due to their probabilistic nature and consequent uncertainties^10,11^. No explicit action plans could have been developed on the basis of those forecasts and the conservative decision making environment further impeded their use^10–12^. The debate about the value of seasonal forecasts already started in the 1970s when the provision of seasonal climate forecasts emerged^13^, and still continues, as the optimal use and value of these forecasts is still unclear^9,10,14^. To implement and foster the usage of seasonal forecasts, the concrete economic benefits need to be demonstrated when decisions and actions are forecast-based. Uncertainties have to be accounted for and economically beneficial probability thresholds for decisions need to be developed^15^. This would allow decision makers to understand the potential benefits of using probabilistic seasonal forecasts.

The assessment of the potential economic value (*PEV*)^16–18^ is a direct way to achieve action recommendations based on forecast probabilities without having detailed operational information and interaction with a decision maker. It is a superordinate base for decisions that incorporates a neutral decision environment (no risk aversion) with costs and losses that arise from taking forecast-based action, and a valuation of the decisions based on relative economic savings^16^. For water management, the estimation of costs and losses associated with decisions made at reservoirs and the consideration of a decision’s economic consequences should be key approaches for dam operations, facilitating access to the economic value approach. Most studies about the economic value of forecasts only involve theoretical examples without quantification of the expected costs and losses, or used the economic value as a further measure to assess the goodness of the seasonal forecasts^18–22^. Monetary values related to forecasts are only provided by few studies, and not in the direct context of the potential economic value assessment^23,24^. Regarding the energy production at reservoirs, costs and losses of dam operations can be specified quite accurately for use in this approach. With energy production at dams playing a major role in many countries^4^, a possible optimization or improvement of the dam operations by the usage of seasonal forecasts could provide a huge benefit, including proactive disaster planning.

The arising benefits when basing early action on hydrometeorological seasonal forecasts for extreme events like drought, very warm and very wet months at forecast horizons up to seven months ahead will be demonstrated for seven selected climate-sensitive, semi-arid, and in parts highly-managed river basins including mountainous and flat areas with extents from tens of thousand to millions of square kilometers. These are the river basins of São Francisco with an artificially created extension (ESF) in Northeast Brazil, of Catamayo-Chira (Chira) in South Ecuador and North Peru, of Karun in Southwest Iran, of the Nile tributaries Tekeze-Atbara (Atbara) and Blue Nile in Ethiopia and Sudan, as well as of Niger and Volta in West Africa. Hit by several severe droughts and floods during the last years^7,25–27^, all these semi-arid study regions depend on sustainable multipurpose water resources management. Their severe vulnerability to climate variability can be explained by a combination of high shares of population living in the especially dry tailwaters of the basins^28^, high dependencies on irrigation or rain-fed agriculture and on hydroelectric power supply by large dams^4^, together with a projected aggravation of climate change signals with higher variability of seasonal precipitation^29^ and prolonged, more intense droughts with intermittent heavy rainfalls^6,30,31^.

For the assessment of economic value, we use the latest seasonal forecast product SEAS5 by the European Centre for Medium-Range Weather Forecasts (ECMWF) at a resolution of 35 km^32^. Providing the main input for water management, we focus on the PEV analysis of precipitation and temperature forecasts of SEAS5. The evaluation method and the selection of analyzed extreme events and evaluated quantities based on commonly used drought indices and separate probability distributions for reference and forecast data allow for the application of raw, uncorrected seasonal forecasts.

With this analysis, we reveal time horizons across which economic benefits exist, and evaluate differences between the considered regions and extreme events. For an in-depth example for the Upper Atbara Dam in Sudan, we calculate the incurred economic consequences of reservoir operations adapted to drought conditions. For water management policies, we further suggest forecast probability thresholds for early action plans based on criteria for minimizing the expenses (expense minimization) and maximizing the number of hit events (event maximization).

## Results

### Increasing frequency of extreme events in semi-arid regions

**Figure 1:**
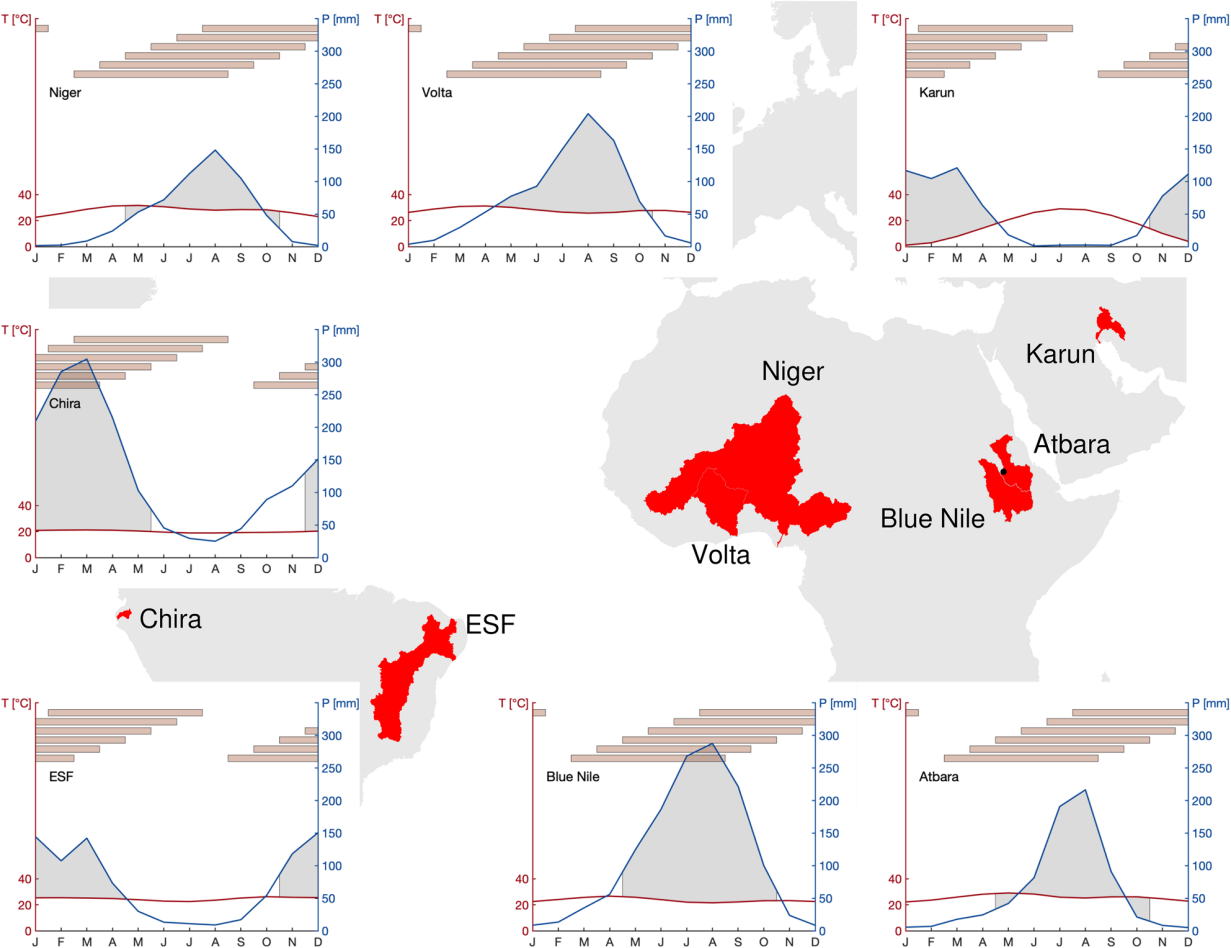
Map of basin areas and climate diagrams with temperature and precipitation. For the seven semi-arid river basins from West to East: Chira and Extended São Francisco (ESF) in South America, Niger and Volta in West Africa, Blue Nile and Atbara in Northeast Africa, and Karun in West Asia. Basin sizes range from ${\mathcal {O}}(10^{4})$ $\hbox {km}^2$ to ${\mathcal {O}}(10^{6})$ $\hbox {km}^2$, with basin altitudinal differences of less than 500 m to about 4000 m. The location of the Upper Atbara Dam Complex is shown with a black dot on the map. The grey shaded area in the climate diagrams defines the main four months of the rainy season, plus the preceding and succeeding month, used for the analysis as “rainy season”. The red bars denote the six-month aggregation periods of a drought index for the analysis during the respective rainy seasons. Coastlines in the map originate from the Global Self-consistent, Hierarchical, High-resolution Geography Database (GSHHG)^48^. Basin boundaries were produced with the HydroSHEDS dataset^49^ and modified for consistency with their respective definitions by local authorities and stakeholders in the study regions.

All semi-arid study basins in South America (Chira, Extended São Francisco), Africa (Niger, Volta, Atbara and Blue Nile) and West Asia (Karun) are characterized by a distinct rainy and dry season (Fig. 1). Using the reference data of ERA5 reanalyses, peak monthly precipitation ranges between 120 and 300 mm. Monthly temperatures are rather constant, except for the Karun basin with a pronounced annual cycle of about 30 $^{\circ }$C. For consistency in the following analysis, the rainy season is defined as the main four months of the rainy season, plus the preceding and succeeding month (Fig. 1).

**Figure 2:**
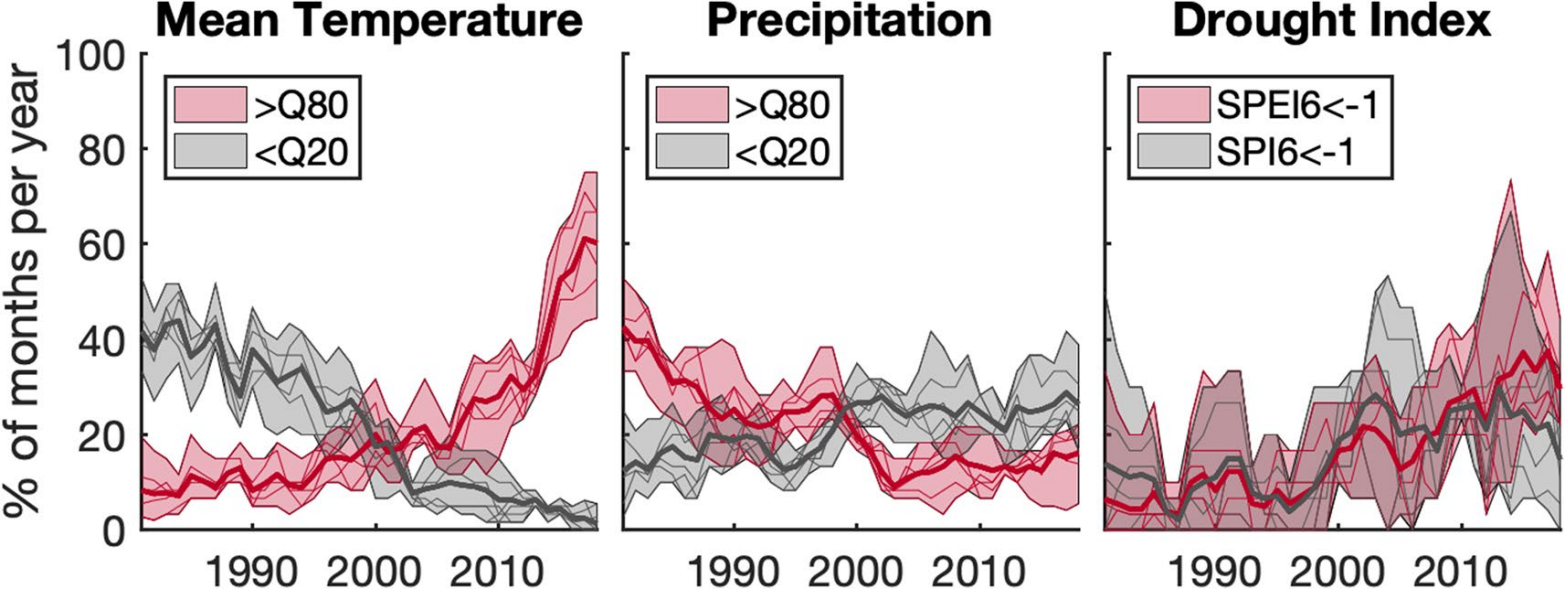
Relative frequency of climate variables from 1981–2018. From left to right the relative frequency of ERA5 mean temperature (*T*, left), total precipitation (*P*, middle) and drought index (SPEI, right) exceeding the respective quantile event thresholds (< Q20, > Q80,$<-1$) are shown for the basin-averages of Extended São Francisco, Niger, Volta, Atbara, Blue Nile and Karun. The thick solid lines denote the mean relative frequency over the 6 basins, and the shaded areas encompass the thin solid lines for the 5-year moving averages of relative frequencies of the individual basins.

For the occurrence of extreme events from 1981-2018, strong signals of climate change are observed across the study regions (Fig. 2). On average, the relative frequency of very warm $T>$ Q80 (cold, $T<$ Q20) months increases (decreases). The trend for very wet months ($P >$ Q80) is negative, while the frequency of six-month droughts indicated by the indices of standardized precipitation (SPI6) and of standardized precipitation and evapotranspiration (SPEI6) smaller than -1 shows a positive trend. The annual frequency range of individual river basins (shaded areas in Fig. 2) is largest for the drought indices, illustrating the large variability among the basins with respect to droughts. All trends in the basin-averaged relative frequency of extreme events shown in Fig. 2 are significant at a significance level of $\alpha =0.05$ (non-parametric Mann-Kendall test, see Supplementary Information). Some trends of individual basins may not be significant. We excluded Chira in this trend analysis of climate extremes in Fig. 2, as not a single trend is found to be significant (also see Supplementary Information).

**Figure 3:**
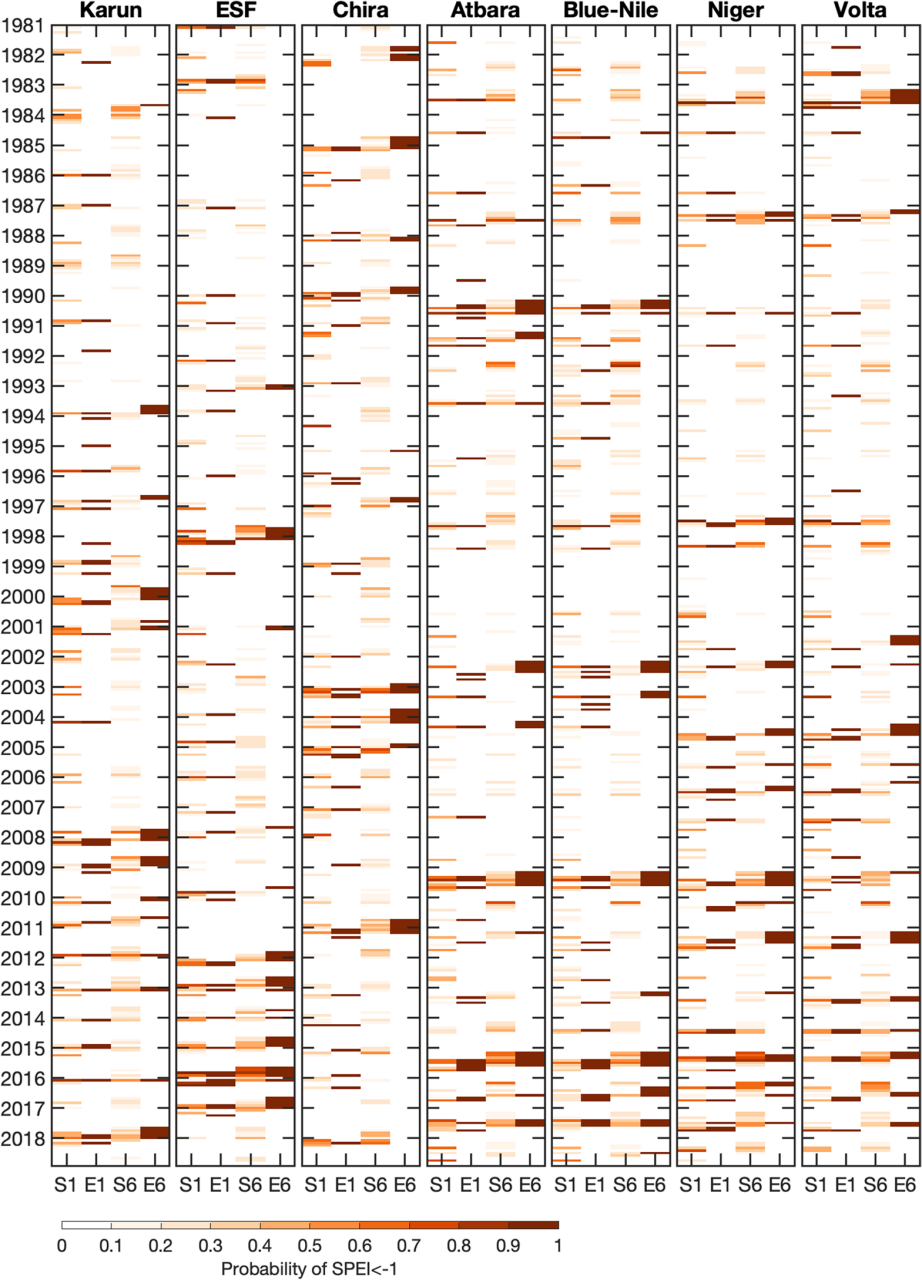
Forecast probability and occurrence of short- and long-term drought. The probability of SPEI1 $<-1$ (1) and SPEI6 $<-1$ (6) for SEAS5 (S) and ERA5 (E) are provided for the rainy seasons of the seven semi-arid regions from 1981-2018. The confinement of the analysis to the basins’ rainy seasons allows a simplified overview of drought events and respective forecast probabilities when droughts are mainly driven by rainfall shortage. The used time periods for aggregation of SPEI6 are depicted with the red bars in Fig. 1.

The occurrence of extreme events in reanalyses is shown together with the respective probability of seasonal (re-)forecasts SEAS5 from 1981–2018 in Fig. 3, for the example of drought conditions (over one month: SPEI1 $<-1$, and over six months: SPEI6 $<-1$) during the basins’ rainy seasons. An increasing occurrence and accumulated SEAS5 probabilities of drought conditions can be identified in the last decade (except for Chira). Here, special emphasis can be placed on the 2011–2017 period of long-term drought conditions (SPEI6 $<-1$ for ERA5) in ESF, also forecast with probabilities of up to 80 and 90% (SPEI6 $<-1$ for SEAS5), during all included rainy seasons. However, lower forecast probabilities of around 40% are also present. For Niger and Volta, the period of 1981-2001 is mainly dominated by three drought events in 1983, 1987 and 1997; after 2001 both long-term and short-term drought events become more frequent and are frequently forecast. For all sub-Saharan African basins between 2015 and 2017, an accumulation of occurred drought periods and elevated forecast probabilities can be identified. Long-term drought conditions (SPEI6) in the study basins, e.g. for Karun in 1999–2001, 2007–2010 and 2018/2019, for the sub-Saharan African basins in 2009 and 2015, and for Chira from 2002–2005, were forecast with variable probabilities ranging from 10 to 90%. A strong variability in monthly rainy season’s precipitation can be observed in the ESF (1990–1994, 2002–2007) and Karun (1985–1987, 1991–1999, 2010–2016) basins with various single months with SPEI1 $<-1$. For the other basins in Africa, in contrast, short-term and long-term drought conditions coincide to a higher degree, suggesting less variability within single months of the rainy season.

### Economic benefit of forecast-based action for extreme events

**Figure 4:**
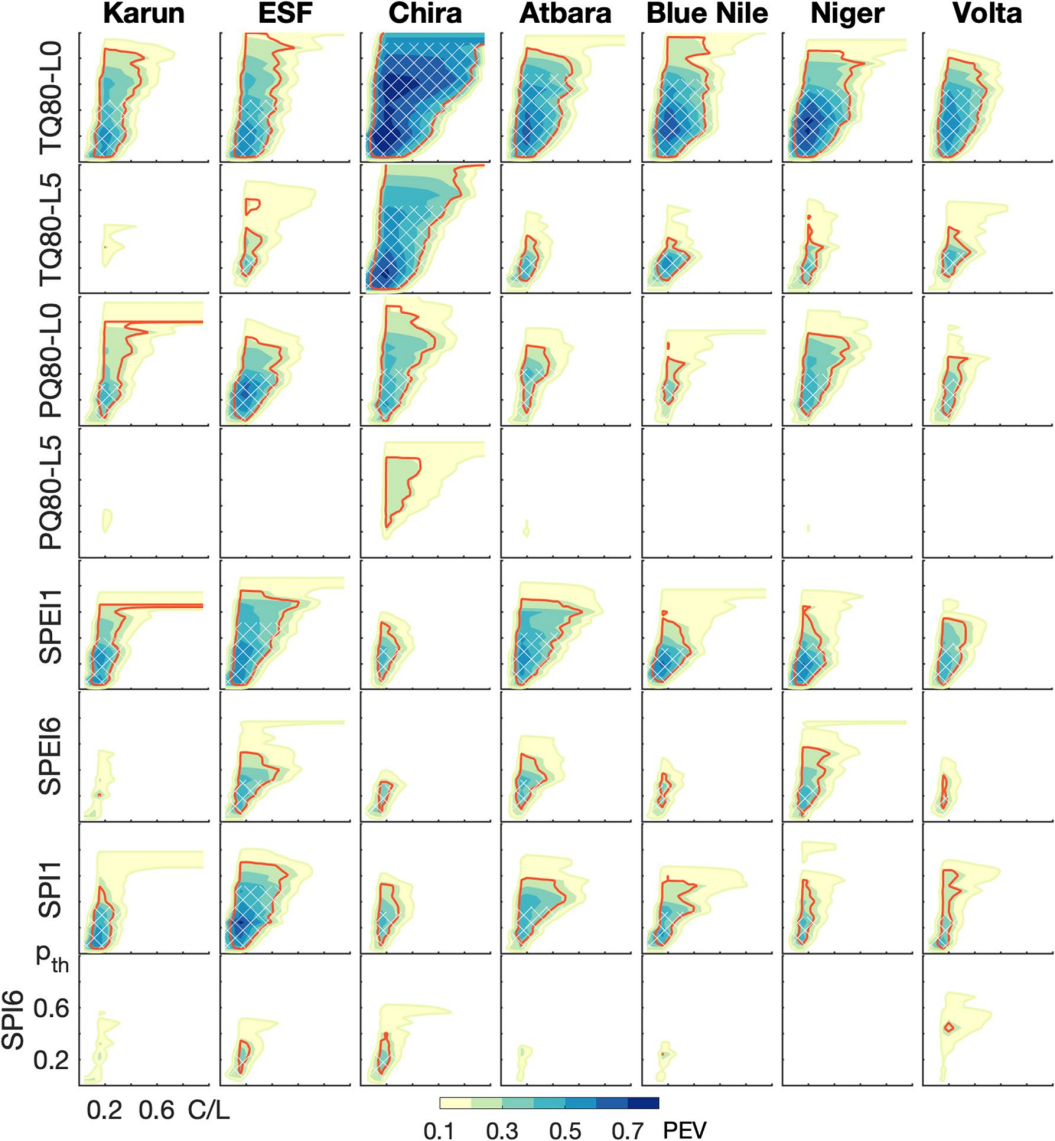
Potential economic value for forecast-based action as a function of probability threshold and user cost-loss ratio. The filled contours denote the potential economic value *PEV* of SEAS5 with reference ERA5 for extreme events of the seven river basins for rainy seasons of the hindcast period 1981-2016. The extreme events include $T>Q80$ and $P>Q80$ for leadmonths 0 (L0) and 5 (L5), as well as SPEI $<-1$ and SPI $<-1$ for aggregation scales over one (SPI1/SPEI1) and 6 months (SPI6/SPEI6). Only valuable conditions are color-coded, i.e., $PEV>0.1$. The uncertainty of the estimation of *PEV* is assessed with bootstrap resampling, with the red contour indicating where 90% of bootstrap samples give a $PEV>0.1$. The hatched area further represents where $PEV>0.1$ and the hit rate $H>0.5$ to base the choice of $p_{\mathrm {th}}$ on the event maximization criterion.

**Figure 5:**
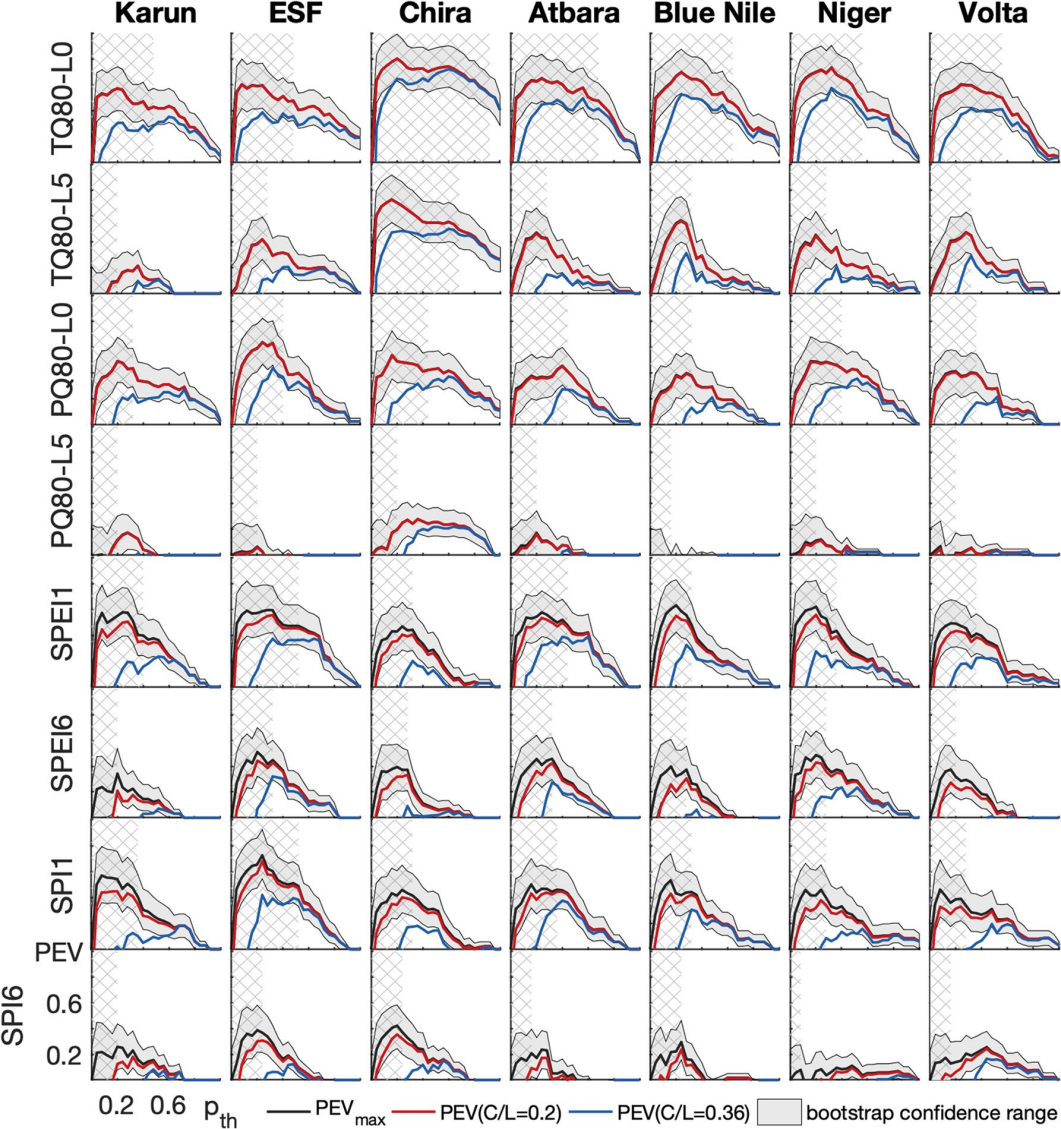
Maximum potential economic value as a function of probability threshold. $PEV_{max}$ of SEAS5 with reference ERA5 is depicted for extreme events of the seven river basins for rainy seasons of the hindcast period 1981-2016 (black line). The uncertainty of the estimation of $PEV_{max}$ is assessed with the confidence range between the 10% and 90% quantiles of bootstrap resampling (shaded area). The red and blue lines denote the *PEV* for cost-loss ratios of 0.2 and 0.36, respectively. The extreme events include $T>Q80$ and $P>Q80$ for leadmonths 0 (L0) and 5 (L5), as well as SPEI $<-1$ and SPI $<-1$ for aggregation scales over one (SPI1/SPEI1) and 6 months (SPI6/SPEI6). The cross-hatched area further represents where the hit rate $H>0.5$ to base the choice of $p_{\mathrm {th}}$ on the event maximization criterion. Note: From its definition, $PEV_{max}$ represents the *PEV* when the cost-loss ratio *C*/*L* is equal to the climatological frequency of the event (see Equation 2), as in this special case both options of never or always acting create the same expense. Climatology can not advise the decision maker and any forecast is of maximum value. The climatological frequency of a Q80-event and SPI/SPEI $<-1$ is 0.2 and 0.159, respectively. Therefore, $PEV(C/L=0.2)$ (red line) and $PEV_{max}$ (black line) for the Q80-events overlap and accordingly cannot be seen separately.

The comparison of forecast probabilities and drought occurrences in Fig. 3 already demonstrated the complexity of deciding whether an event was forecast or not, and the need to identify a probability threshold $p_{\mathrm {th}}$ with which this decision is made. For the seven semi-arid river basins, the potential economic value *PEV* is shown for the extreme events of very warm months ($T>$ Q80), very wet months ($P>$ Q80), and for drought months (SPEI $<-1$ and SPI $<-1$) during the rainy seasons as a function of the cost-loss ratio *C*/*L* and $p_{\mathrm {th}}$ in Fig. 4. Per event, two lead times, i.e., for how many months ahead the forecast is valid (Lead 0: current month ahead, Lead 5: six months ahead), or two aggregation scales (SPI1/SPEI1: one month, SPI6/SPEI6: six months), are provided. Figure 4 includes all information needed for the choice of a beneficial $p_{\mathrm {th}}$ for each *C*/*L* considering the two criteria of expense minimization and event maximization. For both criteria, the range of $p_{\mathrm {th}}$ with maximum *PEV* of each *C*/*L* is relevant; however, the event maximization criterion further includes the condition that the highest possible number of extreme events should be caught by preventative early action, i.e., a maximization of the hit rate *H*. Here, we suggest $H>0.5$ as the lowest threshold for this criterion to always ensure a higher hit rate than miss rate of an event ($H>0.5$ is cross-hatched in Figs. 4 and 5). This therewith involves a certain degree of risk aversion of the decision maker. For the example of a warm extreme event ($T>$ Q80) with Lead 0 in the Karun basin and for expense minimization, a user with $C/L=0.4$ would identify probability thresholds between 0.45 and 0.85 as a beneficial trigger range. However, for event maximization, the same user ($C/L=0.4$) would need to use $0.15<p_{th}<0.45$ when only including potential economic values with high hit rate ($H>0.5$). When further considering the uncertainty of estimation of *PEV*, i.e., the robustness of the choice of $p_{\mathrm {th}}$ (red line in Fig. 4, where 90% of bootstrap samples give a $PEV>0.1$), a beneficial range of $p_{\mathrm {th}}$ for the user with $C/L=0.4$ could only be provided for the expense minimization criteria. For the event maximization criteria, this user would be well-advised to base decisions on the climatological strategy. In this case for $C/L=0.4$, this would be never to act as *C*/*L* is smaller than the climatological frequency of the event.

For all study regions, actions based on short forecast horizons (Lead 0, SPEI1/SPI1) possess higher potential economic values (*PEV*) and larger ranges of possible user situations and beneficial probability thesholds (ranges of *C*/*L* and $p_{\mathrm {th}}$ with $PEV>0.1$) than actions based on the respective long-range forecasts (Lead 5 and aggregation periods over 6 months SPEI6/SPI6). Provided that action protocols are already established, the SEAS5 seasonal forecasts released on the 6th of each month can thus provide promising decision support for monthly action adaptations in addition to medium-range weather forecasts. Later release dates would diminish the usefulness of high-valued Lead-0 or SPEI1/SPI1 forecasts. The actual range of users (*C*/*L*) that can benefit from forecast-based action depends on the region and $p_{\mathrm {th}}$: Among the basins, Chira provides the largest ranges of possible users and of beneficial probability thresholds for the warm ($T>$Q80) and wet ($P>$Q80) extreme events of both shown lead times. For drought forecasts of SPEI1 and SPI1, however, the ESF and Atbara basins show the largest areas of *PEV*. For Karun, mainly forecast horizons of one month (Lead 0 and SPEI1/SPI1) can offer economic value to users. For all regions, basing action on temperature forecasts ($T>$ Q80) of Lead 0 is beneficial for a wide range of $p_{\mathrm {th}}$. The regions have in common that very limited *PEV* can be achieved by action based on Lead 5 forecasts of $P>Q80$ and for forecasts of SPI6 $<-1$. Generally, the consideration of sampling uncertainty in the *PEV* estimation (red contour in Fig. 4) further reduces the ranges of users and probability thresholds with value. The comparison of the two drought indices SPEI6 and SPI6 for long-term drought events reveals that the inclusion of temperature in SPEI can increase the value of seasonal forecast-based action for most basins. Despite limited ranges of users and probability thresholds, *PEV* values above 0.4 are still possible for SPEI6 $<-1$, allowing a longer-term decision-support for drought conditions over the rainy seasons.

Considering the maximum potential economic value $PEV_{max}$ over all users (black solid line in Fig. 5), the event maximization criterion (cross-hatched) clearly cuts down the range of beneficial $p_{\mathrm {th}}$ towards lower thresholds. Here, the definition of the maximum potential economic value as the difference between hit and false alarm rate ($PEV_{max}=H-F$) further reveals that the hit rate can still be higher than the false alarm rate when $PEV_{max}>0$, however, the event maximization criterion of $H>0.5$ (hit rate higher than miss rate) may no longer be valid. Exemplary for individual cost-loss ratios, we chose $C/L=0.36$ and 0.2, that are also relevant for the real case study at the Upper Atbara Dam of the next section (also see Table 1). For $C/L=0.36$ (blue line in Fig. 5), the effects of the different criteria on the choice of $p_{\mathrm {th}}$ become even more pronounced: Although the maximum *PEV* for this *C*/*L* would be achieved at higher threshold probabilities (expense minimization), e.g. around $p_{th}=0.6$ for wet extreme events of Lead 0 in Chira, or around $p_{th}=0.4$ for warm extreme events of Lead 5 in ESF, lower $p_{\mathrm {th}}$ around 0.35 and 0.2, respectively, would be preferable in the same examples for event maximization. For other events and basins, like for the Lead 5 warm extreme events in Blue Nile, or for drought conditions SPEI1 $<-1$ in Niger, the probability threshold with maximum *PEV* for the cost-loss ratio of 0.36 does not change with the inclusion of the event maximization criterion. For some regions and events, no beneficial $p_{\mathrm {th}}$ might even be found under the event maximization criterion for higher cost-loss ratios like $C/L=0.36$, though showing potential economic value around 0.2 at higher $p_{\mathrm {th}}$. This is for example the case for Chira and $P>$Q80 for Lead 5, and for Volta and SPI1/6 $<-1$. For lower cost-loss ratios, e.g. $C/L=0.2$ (red line in Fig. 5), this difference in the two criteria is less evident, as higher *PEV* values occur at lower probability thresholds for the extreme events, i.e., beneficial forecast-based action for those users is already provided at higher forecast uncertainty.

**Table 1:** Contingency table for forecast-based action. For the four action-event scenarios, the economic consequences are shown. For the case study of drought and standard reservoir operations at the Upper Atbara Dam in Sudan, the costs and losses for different outcomes of the decision model without operation restrictions (upper line of reservoir operations) and with sediment sluicing (lower line of reservoir operations) are provided. The resulting ratio of costs for action (*C*) and avoidable losses (*L*) for a drought operation without restrictions and with sediment sluicing are 0.37 and 0.19, respectively.

	No extreme **event **$X_{crit}$ is not exceeded	Extreme **event** occurs $X_{crit}$ is exceeded
Early** action** based on forecast $p_{\mathrm {th}}$ is exceeded	*False alarms (f)* Futile action (*C*) Unnecessary costs for action	*Hits (h)* Valuable action (Part of) losses avoided, costs for action ($L_{event} - L+C$)
**Drought reservoir operation**	$$C = 6.0\cdot 10^6~$$US$ $$\hbox {a}^{-1}$$ $$C = 0.3\cdot 10^6~$$US$ $$\hbox {a}^{-1}$$	$$L_{drought}-L+C = 20.9\cdot 10^6~$$US$$$\hbox {a}^{-1}$$$$\rightarrow$$$$L = 16.2\cdot 10^6~$$US$ $$\hbox {a}^{-1}$$ $$L_{drought}-L+C = 16.4\cdot 10^6~$$US$ $$\hbox {a}^{-1}$$$$\rightarrow$$$$L = 1.6\cdot 10^6~$$US$$$\hbox {a}^{-1}$$
No early **action**$p_{\mathrm {th}}$ is not exceeded	*Correct rejects (r)* Valuable inaction No costs or losses (-)	*Misses (m)* Erroneous inaction No losses avoided ($L_{event}$)
**Standard reservoir operation**	0 0	$$L_{drought}=31.1\cdot 10^6~$$US$ $$\hbox {a}^{-1}$$ $$L_{drought}=17.7\cdot 10^6~$$US$ $$\hbox {a}^{-1}$$

**Figure 6:**
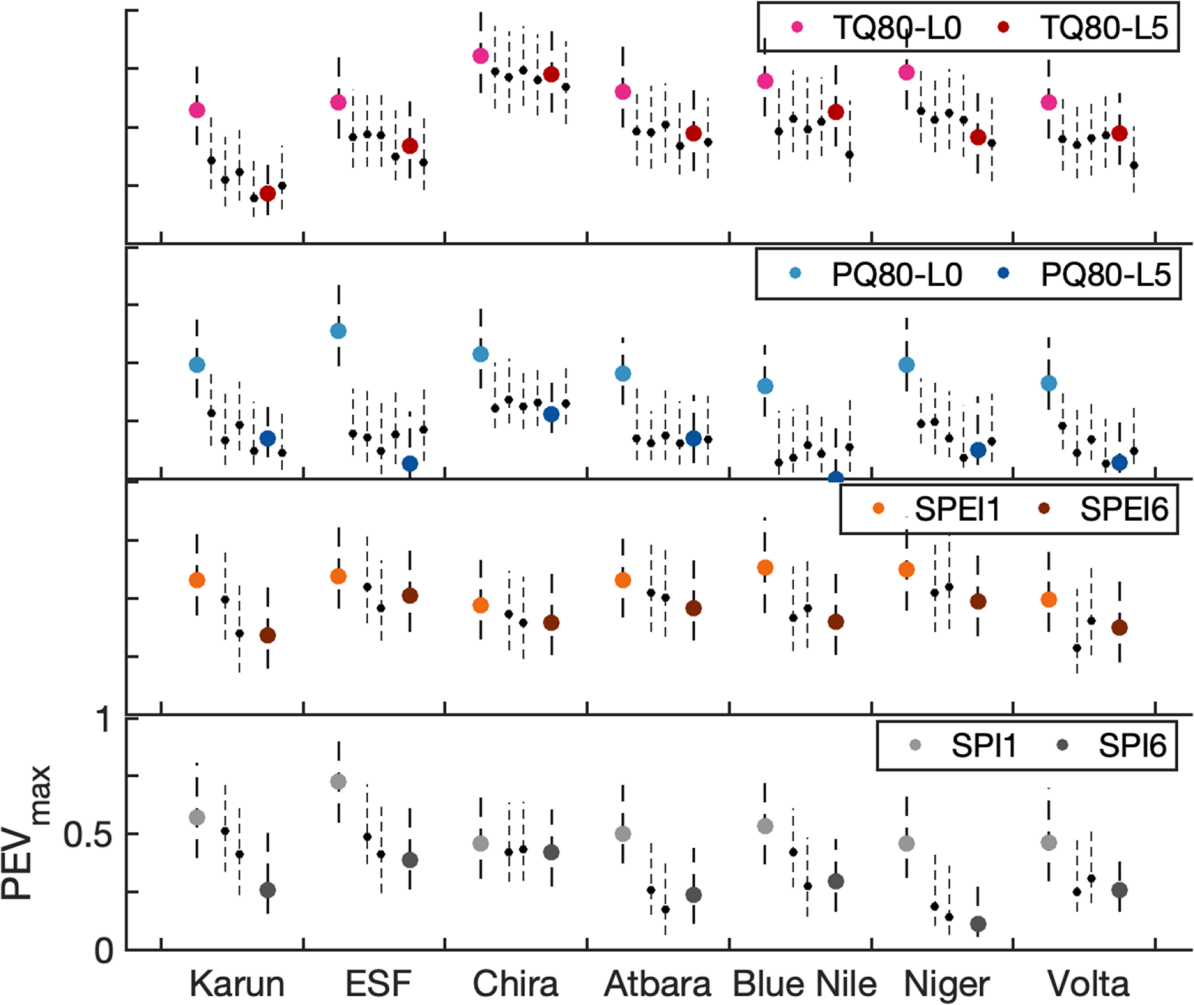
Maximum potential economic value for forecast-based action. $PEV_{max}$ of SEAS5 with reference ERA5 is shown for the seven river basins for rainy seasons of the hindcast period 1981-2016. Colored dots represent the fully evaluated $PEV_{max}$ for the events of $T>Q80$ and $P>Q80$ for leadmonths 0 (L0) and 5 (L5), and for the events SPEI $<-1$ and SPI $<-1$ for aggregation scales over one (SPI1/SPEI1) and 6 months (SPI6/SPEI6). The uncertainty of the estimation of $PEV_{max}$ is assessed with the confidence range between the 10% and 90% quantiles of bootstrap resampling (dashed line). $PEV_{max}$ is also given for temperature and precipitation of leadmonths 1-4 and 6, and for drought events of scales over 3 and 4 months (black dots) with respective bootstrap confidence ranges.

Further summarizing $PEV_{max}$ over all probability thresholds, the effect of lead time and aggregation scale on the value of forecast-based action for the different extreme events can be studied (Fig. 6). For most regions, forecast-based action for warm extreme events ($T>$ Q80) is most valuable and maintains higher maximum potential economic value with increasing lead time than those for wet extreme events ($P>$ Q80). For warm extreme events, Lead 0 forecasts attain maximum potential economic values between 0.55 and 0.8, whereas for wet extreme events, $PEV_{max}$ of Lead 0 forecasts ranges between 0.38 and 0.64. The uncertainty of $PEV_{max}$ estimation is taken into account with the bootstrap confidence range (dashed lines in Fig. 6), e.g., specifying a range of $0.64<PEV_{max}<0.98$ for warm extreme events of Lead 0 in Chira with the 10% and 90% of bootstrap percentiles. Except for Chira with a smooth decrease of maximum potential economic value for warm extreme events with lead time, all regions show an abrupt decline of $PEV_{max}$ from Lead 0 to Lead 1. For most regions, this strong drop is larger than the decline of $PEV_{max}$ from Lead 1 to Lead 5. The most abrupt drop of $PEV_{max}$ is evident for wet extreme events in ESF from 0.75 of Lead 0 to 0.24 of Lead 1. For drought conditions, the decline with longer aggregation scales is less pronounced than the decline with single lead times for the wet extreme, as still higher-valued months of lower forecast horizon are included in SPI/SPEI. In basins like ESF, Blue Nile and Volta, where $PEV_{max}$ of Lead 5 for wet extreme events is close to zero, longer aggregation scales of the dry extreme (SPI6 $<-1$) still attain values between 0.24 and 0.38. For SPEI the decrease of $PEV_{max}$ with longer aggregation scales more resembles the one of the warm extreme with increasing lead time, with relatively high maximum potential economic values between 0.28 and 0.54 still for longer aggregation scales. The inclusion of temperature attaining high values still at longer lead times in the drought index of SPEI allows for higher $PEV_{max}$ at long aggregation scales relative to the solely precipitation based drought index of SPI.

### Water level management and energy production during drought reservoir operations: the Upper Atbara Dam case

The calculation of the cost-loss model for drought events is demonstrated for the water level management and energy production at the Upper Atbara Dam in Sudan. Though having two dam operation examples including and neglecting sediment restrictions for a reservoir drawdown, this study cannot suggest completeness of all relevant processes, decisions and measures. However, it is a demonstration of application of the cost-loss model and the above generated results.

**Figure 7:**
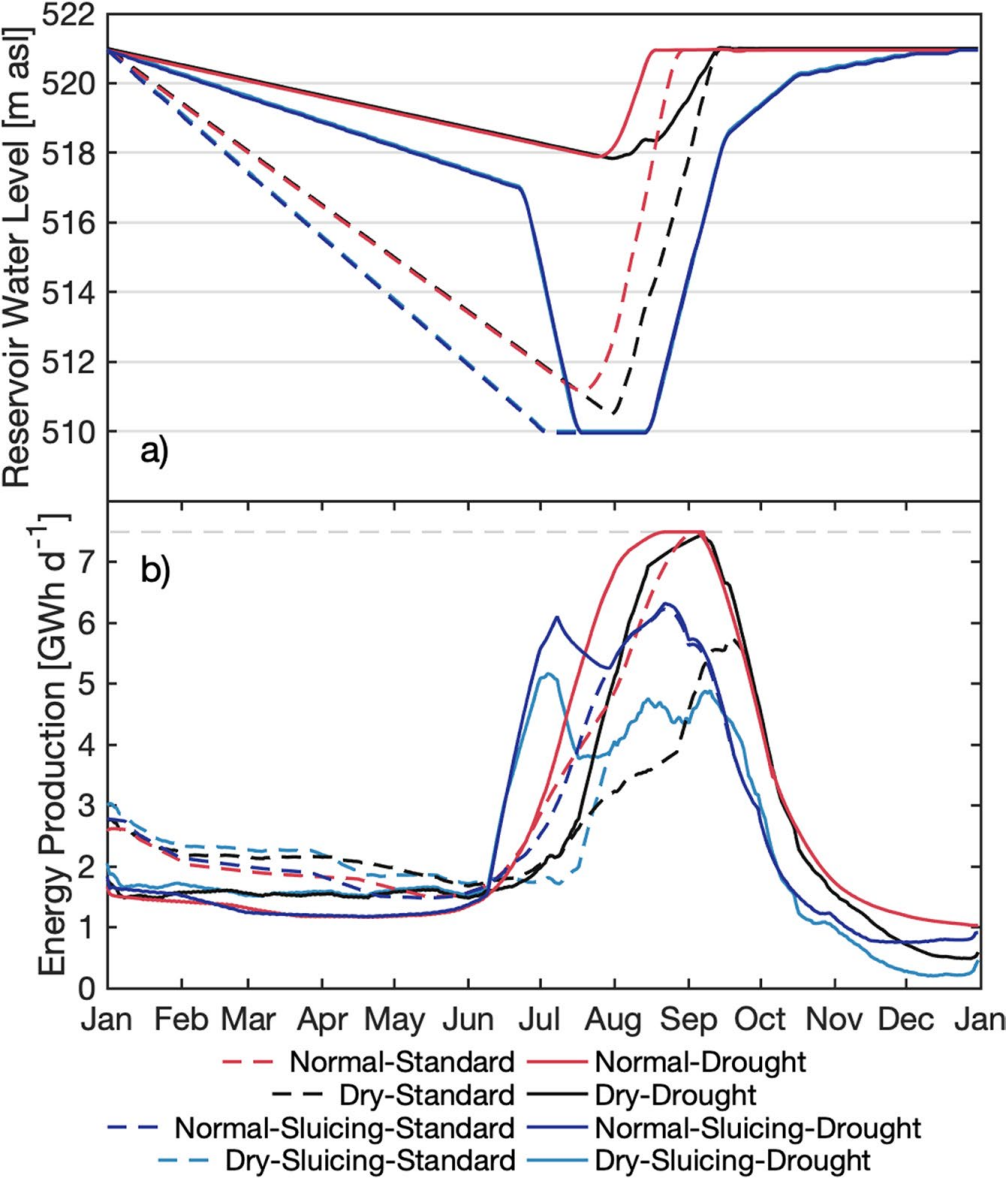
Reservoir management at the Upper Atbara Dam Complex in Sudan. **(a)** Reservoir water level (shown as 5-day moving average) and **(b)** energy production (shown as 30-day moving average) for a rainy season in a normal year without operation restrictions (red lines, “Normal”), for a rainy season under drought conditions without restrictions (black lines, “Dry”), for a rainy season in a normal year including sediment sluicing (dark blue lines, “Normal-Sluicing”), and for a rainy season under drought conditions including sediment sluicing (light blue lines, “Dry-Sluicing”, in **(a)** identical to dark blue line). The dashed lines denote reservoir management with standard reservoir operations. The solid lines show the altered reservoir management for drought conditions, i.e., having prior knowledge of drought. In **(b)**, the light gray dashed line marks the maximum possible energy production (7.5 GWh $\hbox {d}^{-1}$), i.e., the maximum reservoir capacity. Any additional inflow would need to be passed through the spillway, without the usage for energy production.

Having no operation restrictions on the Upper Atbara Dam, water level changes are governed only by a reservoir operation to maximize electrical energy generation. The standard operation at the Upper Atbara Dam with the peak of the rainy season in July/August is a gradual lowering of the reservoir water level from the full supply level (FSL) at 521 m asl by about 10 m from January on to be able to take advantage of the stored reservoir volume and to absorb the inflow during the rainy season (red dashed line in Fig. 7a). As the expected inflow comes, the reservoir is filled again to the FSL.

In a dry year, the water level curve differs in July and August when the operator first still awaits the maximum inflow to come, but needs to fill the reservoir until September to FSL (black dashed line in Fig. 7a). The latter requires a high amount of the inflow for filling as the expected peak inflow was absent. Compared to a normal year, this results in a strong decline by about 30% in energy production from July to September (black dashed line in Fig. 7b).

With prior knowledge of drought conditions during the rainy season, i.e., applying drought operations, the reservoir is kept at a higher water level with higher heads (black solid line in Fig. 7a), lowering the energy production in the first phase by about 25%, but allowing for considerably higher energy production (+40%) in the second phase compared to the standard operations (black solid line in Fig. 7b).

If a normal year was falsely operated as a dry year, e.g., a seasonal forecast falsely predicted drought conditions for the rainy season of a normal year, the reservoir with the reduced water level by only 3 m in the first phase is not able to absorb the normal amount of inflow (red solid line in Fig. 7a). The reservoir operates at its maximum capacity and part of the inflow needs to be passed through the spillway, without the usage for energy production (red solid line in Fig. 7b).

For the decision whether to modify the reservoir operation, i.e., to take preventive action for a coming drought event or not, the decision-maker needs to evaluate the economic consequences. The losses ($$L_{drought}$$), when the drought occurs but no action is taken, are calculated from the yearly difference of the earned money applying standard operations during a normal year (Normal-Standard, $$203.5\cdot 10^6~$$US$ $$\hbox {a}^{-1}$$) and during a dry year (Dry-Standard, $$172.4\cdot 10^6~$$US$ $$\hbox {a}^{-1}$$), and amount to $$31.1\cdot 10^6~$$US$ $$\hbox {a}^{-1}$$ (Table 1). The costs $$C=6.0\cdot 10^6~$$US$ $$\hbox {a}^{-1}$$ arise from the yearly difference of the earned money during a normal year with standard operations (Normal-Standard) and a normal year that is falsely treated as a drought year (Normal-Drought, $$197.5\cdot 10^6~$$US$ $$\hbox {a}^{-1}$$). For correctly-taken action, costs *C* and unavoidable losses $$L_{drought}-L$$ of in total $$20.9\cdot 10^6~$$US$ $$\hbox {a}^{-1}$$ incur, determined from the difference of earned money in a normal year with standard operations and of a dry year with drought operations (Dry-Drought, $$182.6\cdot 10^6~$$US$ $$\hbox {a}^{-1}$$). *L* are the potential losses protected by the action, and result in $$16.2\cdot 10^6~$$US$ $$\hbox {a}^{-1}$$. The characteristic cost-loss ratio *C*/*L* of preventive drought operations for the Upper Atbara Reservoir, i.e., the cost of acting as a fraction of the potential loss prevented by the action, thus is 0.37.

With the closest resolvable $C/L=0.36$, the resulting maximum *PEV* of 0.4 (expense minimization) for drought events of SPEI1 in the Atbara basin is obtained for $p_{th}=0.6$ (Fig. 5). Considering the event maximization criterion, a lower threshold around $p_{th}=0.32$ is beneficial, however with lower *PEV* of 0.35. For the larger timescales of SPEI6, both expense minimization and event maximization criteria result in most beneficial forecast-based action for $p_{th}=0.32$ with $PEV=0.27$.

At high sediment-trapping dams in semi-arid regions often sediment sluicing operations during the peak of the rainy season are required. Here, the reduction of the reservoir water level to the minimum operation level (MOL) during high inflows increases the bottom shear stress maintaining incoming sediment in suspension and remobilizing deposited sediment with only limited or no reservoir deposition.

At the Upper Atbara Dam, the MOL at 510 m asl is usually held for six weeks during July and August and may be extended if inflows still exceed 3000 $\hbox {m}^3$ $\hbox {s}^{-1}$. In general, the operation restriction by sluicing reduces the energy production in a normal year by about 13% due to lower heads (dark blue dashed line in Fig. 7b).

In a dry year, the same operation restriction for sluicing with a steady drawdown to MOL is performed (light blue dashed line in Fig. 7a). As inflows are lower, about 23% less energy is produced from July until September compared to a normal year (light blue dashed line in Fig. 7b).

With the application of drought operations, i.e., having prior knowledge of drought, the water level is first more slowly lowered, also reducing the produced energy in this phase by 14% (light blue solid line in Fig. 7). Then, the water level is more rapidly lowered down to the sluicing level, thus allowing for a higher energy production (+17%) from July until September compared to standard sluicing operations due to higher heads. As the expected inflow is low and carries less sediments than high inflow, the period of sediment sluicing is reduced to four instead of six weeks.

Falsely applied drought reservoir operations in a normal year first show a lower energy production and then a higher energy production (dark blue solid line in Fig. 7b). The yearly difference in energy production between the standard (Normal-Sluicing-Standard, $$177.3\cdot 10^6$$ US$ $$\hbox {a}^{-1}$$) and drought reservoir operation (Normal-Sluicing-Drought, $$177.0\cdot 10^6$$ US$ $$\hbox {a}^{-1}$$) only amounts to 0.17%.

Similarly to the case without reservoir operation restrictions, the costs and losses for the drought operation including sediment sluicing can be calculated (Table 1). The costs for drought operations amount to $$0.3\cdot 10^6$$ US$ $$\hbox {a}^{-1}$$. The total losses of a dry rainy season $$L_{drought}$$ are $$17.7\cdot 10^6$$ US$ $$\hbox {a}^{-1}$$ (with Dry-Sluicing-Standard: $$159.6\cdot 10^6$$ US$ $$\hbox {a}^{-1}$$), of which $$L=1.6\cdot 10^6$$ US$ $$\hbox {a}^{-1}$$ can be avoided by drought reservoir operations (with Dry-Sluicing-Drought: $$160.9\cdot 10^6$$ US$ $$\hbox {a}^{-1}$$). The resulting cost-loss ratio is 0.19.

With these identified costs and losses (closest resolvable $C/L=0.2$), seasonal drought forecasts of ECWMF SEAS5 generate potential economic values between 40 and 55% for both short (SPEI1) and long (SPEI6) time horizons, and for both the event maximization and expense minimization criteria (Fig. 5). The most beneficial $p_{th}$ is therewith identified at around 30% for high hit rates above 50%.

## Discussion

This study presents how decision-makers can optimize water management by the use of seasonal forecasts and directly obtain robust beneficial probability thresholds triggering preventive action on the chosen criterion of either expense minimization or event maximization. This valuation approach is a tool for user-oriented, economic decision-related verification of probability forecasts, away from the classical approach of evaluating forecasts based on skill scores^33,34^, whose statistics may not be conclusive in a decision making environment. Of course, the classical quality assessment based on skill scores and the economic value assessment should complement each other. However, the aims and advantages behind both approaches are different^35^: The skill/quality approach aims at the improvement of correspondence of forecasting systems with observations as well as the intercomparison of different forecasting systems. It has the advantage of identifying their strengths and flaws with respect to different quality skill measures (e.g., forecast bias, distribution, sharpness). The valuation approach has so far been applied for, e.g., promoting the usage of ensemble forecasts^16^, verification of seasonal forecasts^20,36^, and even for decision-support of preventive humanitarian action^18^. Compared to a quality skill score analysis, the valuation approach as used in our study aims at building customer confidence in forecast products by demonstrating the economic benefit realized by decision-makers through the use of the forecasts. It has the clear advantage of providing decision-support for individual users with an individual choice of beneficial probability thresholds for a special event. A common skill score of forecast quality aggregates the forecast performance over all users (cost-loss ratios) and probability thresholds. For example, $PEV_{max}=H-F$, also known as the Kuipers Skill score^16^, is the maximum economic value over all *C*/*L* and $p_{th}$. In our case, skillful forecasts ($PEV_{max}>0$) for all lead times and scales were found for all considered extreme events (except for Blue Nile $P>Q80$ of Lead 5, Fig. 6), however, the individual cost-loss situation of an action determines the individual value and action trigger. For certain users, skillful forecasts might involve non-positive economic value (Figs. 4 and 5). Considering the aggregation timescale of the drought index SPEI $<-1$, the forecast skill $PEV_{max}$ only decreases slightly with longer timescales (Fig. 6). However, the confident range of $p_{\mathrm {th}}$ and cost-loss situations with $PEV>0.1$ may shrink drastically from SPEI1 to SPEI6 (Fig. 4), demonstrating the necessity of a detailed look at *PEV* for each *C*/*L* and $p_{\mathrm {th}}$ (Fig. 4). Therefore, skillful seasonal forecasts of hydrometeorological events *per se* are not necessarily valuable to a specific decision maker and a considered action.

In our analysis, we used uncorrected, possibly biased forecasts. This is reasonable due to the usage of relative thresholds, i.e., quantiles of the distributions of forecast and reference data separately, instead of absolute values, as well as drought indices of separate forecast and reference distributions. Other studies^37,38^ merged the reference and forecasts data in their calculation of drought indices and therefore required the two datasets to have at least the same mean climate, i.e., they required linear-scaling bias correction of monthly data per lead time. With our separate treatment of distributions of forecast and reference data, mean and distributional biases that would be removed by linear scaling or quantile mapping bias correction are implicitly taken into account. Even ensemble calibration methods^39^ won’t have an effect on the relative quantile values of separate reference and forecast distributions, as they do not change the relative location of the quantile value within the system’s distribution when only mean and standard deviation of the ensemble are changed. Forecast distributions are further used for each lead time and scale separately, diminishing the evident effect of model drift with lead time. Uncorrected raw forecasts can thus be used as long as (1) distributions are separately treated for reference and forecast data, (2) the temporal (e.g. monthly; per lead time) and spatial (basin-averages) resolutions are preserved and (3) no absolute values or event thresholds (e.g., $> 5$ mm) are used. Nevertheless, more sophisticated post-processing measures, such as multivariate bias correction^40^ or even dynamical downscaling^41^ may show an effect on the forecast value.

Regarding the transferability of the results, the catchment size was found to have only a minor impact on the economic value of seasonal forecasts. The results rather seem to be independent of the basin size. For differences in economic value between the basins, general circulation patterns and their predictability may play a larger role (e.g., ENSO temperature effect in Chira, influence of short predictability of westerlies in Karun). Consistently among all considered semi-arid basins that are far apart from each other in South America, Africa and West-Asia and with different dominating weather systems, higher PEV and larger beneficial ranges of probability thresholds and possible user situations were evident for lower forecast timescales than for higher ones (Fig. 4). The warm extreme events ($T>$ Q80) were shown to provide the respective highest values of seasonal forecasts, even for higher lead times (Fig. 6). Similarly, drought forecasts (SPEI) allowed for high *PEV* even at high aggregations scales (SPEI6). This consistency of a high value among all different study regions might indicate a transferability of the results to other regions. Economic benefits could likewise be expected from temperature and drought forecasts of even high forecast horizons up to six months, and from precipitation of at least one month ahead. As in our study regions, certain users may thus still benefit from large forecast horizons. However, further studies still must confirm this validity for other regions of the world.

We provided an in-depth example for water reservoir management at the Upper Atbara Dam in Sudan for energy production as a real-case practical application of the cost-loss decision model. For this example, dam operation standards are based on practical experiences of the dam management agency, however, flexibility of the reservoir manager is also possible when using other fix dates or even when considering energy demand and price. Thus, a wide range of cost-loss ratios can be obtained controlled by different underlying assumptions. Furthermore, the cost-loss situation was focusing on hydropower generation, not yet considering, e.g., costs and losses for irrigation. With this, we provided a straightforward application of the cost-loss decision model for forecast-based action. We further promote the implementation of seasonal ensemble forecasts for concrete water management operations at the Upper Atbara dam. The given example there, though including limitations and being demonstrated for only a specific year so far, showed that up to one half ($$16.2\cdot 10^6$$ US$) of the losses associated with the energy production in a drought year can be avoided if altered management operations are applied.

The increase shown in the frequency of droughts and warm extremes during the last decades together with high population growth rates in semi-arid regions, raise the pressure on multipurpose reservoirs, such as the Upper Atbara Dam. Longer-term sustainable planning several months ahead prior to the next rainy season becomes even more crucial. With rising demands on the water management, switching towards beneficial seasonal-forecast-based early actions saves expenses and aids climate proofing. Consequently, we stress the advantage and necessity of considering seasonal forecasts in hydrological decision making.

## Methods

### Seasonal climate forecast and reference data

SEAS5^32^ is the latest global seasonal forecast system of the European Centre for Medium-Range Weather Forecasts (ECMWF) launched in 2017 including 51 ensemble members. For verification purposes, SEAS5 reforecasts are provided for the period 1981-2016 including 25 ensemble members. Both forecasts and reforecasts are initialized on the first day of each month and provide a forecast horizon of 7 months. Its horizontal resolution is approximately 36 km. SEAS5 is a coupled atmosphere-ocean-sea-ice model that is particularly powerful in the prediction of El Niño Southern Oscillation (ENSO)^32^.

Being aware of the uncertainty of global precipitation reference products, and further requiring intrinsic dependence structure and interaction between, as well as the temporal coincidence and physical consistency of precipitation and temperature, the climate reanalysis product ERA5^42^ is used as reference for both precipitation and temperature. Being system-consistent with SEAS5, ERA5 is offered by the ECMWF with a reanalysis period from 1979 to within 3 months of real time. Historical observations are combined using advanced modelling and data assimilation systems producing global, consistent estimates of climate variables at a horizontal resolution of 30 km.

For this study, monthly area-weighted basin averages of ERA5 and SEAS5 2-m mean temperature and precipitation are used. To preserve the original data and to avoid artifacts due to interpolation methods, basin averages were produced with masks for reference and forecast grids separately. The applied evaluation methods are all based on relative values, i.e., quantiles of the distribution of values per ERA5 and per SEAS5 separately. This approach of separate distributions of reference and forecast data implicitly performs a bias correction of systematic biases in mean and quantile values and therefore does not require additional prior bias correction of the forecast data. Also model biases of the seasonal forecasts, i.e., biases that drift over lead time, are implicitly taken into account by comparing only the same lead times of each month with one another. This implies different distributions and thus quantile values for individual lead times per month making use of the long period of available re-forecasts.

### Drought indices

The standardized precipitation index (SPI) is a drought index based only on precipitation data^43^. The use of SPI assumes that droughts are controlled mainly by the temporal variability in precipitation, neglecting the variability in temperature or potential evapotranspiration (PET).

As the efficiency of drying resulting from temperature anomalies can be as high as that due to rainfall shortage, the standardized precipitation evapotranspiration index (SPEI) also includes PET^44^. Consequently, unlike SPI, SPEI is sensitive to global warming and also indicates drought conditions for increased evapotranspiration due to increased temperature despite of normal precipitation conditions^44^.

Both SPI and SPEI have a multi-temporal nature and their variables of precipitation and water surplus/deficit, respectively, are aggregated at different time scales^43,44^. Unlike in historical analyses, the application of seasonal forecasts suggests the calculation of the multi-month scale of SPI/SPEI in a forecast - not in a retrospective - manner, i.e., SPI6 and SPEI6 of June specify the accumulation from June to November for both reference and forecast. For the calculation of both SPI and SPEI, probability distributions of reference and forecast data are separately treated, i.e., data are not merged, allowing again the use of uncorrected raw forecasts. With the relationship of empirical cumulative probability to the respective aggregated variable of reference and forecast data separately, the associated inverse normal of the respective cumulative probability is estimated. The then derived deviation for a standard normally distributed quantity with zero mean defines the standardized drought indices SPI and SPEI (also see Supplementary Information). In this study, we chose the threshold of SPI/SPEI $<-1$ for the drought analysis, representing all negative anomalies larger than one standard deviation. This encompasses the drought categories of moderate, severe and extreme drought and approximately 15.9% of the data^43^.

### Potential economic value

The benefit from including seasonal climate forecasts in the decision process for water management can be quantified by the potential economic value (*PEV*)^16,17^. In this decision process, actions taken in the water management would be based on seasonal climate forecasts.

For this, we evaluate the performance of the seasonal forecast by combining the outcomes of a binary forecast (hit, false alarm, miss and correct reject) with a cost-loss approach (Table 1). As seasonal forecasts are probabilistic forecasts, i. e., consist of 25 (reforecasts) or 51 (forecasts since 2017) ensemble members, the definition of “the event is forecast” depends on a probability threshold $p_{\mathrm {th}}$. Seasonal forecast-based action will be induced whenever the $p_{\mathrm {th}}$ of the ensemble forecast for an event is exceeded. No action is taken for lower probabilities. The cost-loss ratio *C*/*L* defines the ratio of the cost for action over the avoidable losses by action and is a characteristic of the individual user.

*PEV* is a measure of relative savings. The savings arise from the comparison of the expenses for the forecast-based action to expenses of a reference strategy, the climatological approach ($E_{\mathrm {climate}} - E_{\mathrm {forecast}}$). The climatological approach involves either always acting or never acting, with an optimal course defined by the relation of *C*/*L* and the climatological frequency of the event ${\overline{o}}$: always act if $C/L<{\overline{o}}$, and never act otherwise. The savings $E_{\mathrm {climate}} - E_{\mathrm {forecast}}$ are set into relation with the savings of having a perfect forecast available, i.e., optimal early action due to perfect knowledge of the future weather with which the decision maker acts only when the event was going to occur. This results in:

1 $$\begin{aligned} PEV = \frac{E_{\mathrm {climate}} - E_{\mathrm {forecast}}}{E_{\mathrm {climate}} - E_{\mathrm {perfect}}} \end{aligned}$$

The upper boundary ($PEV=1$) is defined by the savings of a perfect forecast and the lower boundary ($PEV=0$) by the climatological approach. The user benefits from the forecast-based action if $PEV>0$. For $PEV\le 0$, the usage of the climatological approach is more beneficial. Depending on *H* and *F*, *PEV* can be calculated for different *C*/*L* and $p_{th}$:2To estimate the uncertainty of *H*, *F* and *PEV* due to sampling errors of the extreme events, bootstrap resampling is applied^20^ (also see Supplementary Information for more details on the bootstrapping algorithm). The advantage of the bootstrap approach is that no assumption regarding the distribution of the values is required^45^. Here, at the equal size of the original data set, pairs of event-based forecast probability time series and binary reference time series are randomly selected with replacement. This resampling was repeated up to the bootstrap sample size of 1000. To assess the robustness of forecast thresholds^18^, confidence intervals for *PEV* are provided based on the 10 % and 90 % quantiles of the bootstrap samples.

The choice of beneficial $p_{\mathrm {th}}$ can be based on the expense minimization or event maximization criteria^18^. For expense minimization, the range of $p_{\mathrm {th}}$ with highest *PEV* for a specific user (*C*/*L*) is chosen. For event maximization, this is further put under the constraint that *H* should be high to catch as many events as possible and to avoid losses. This event maximization criterion therefore involves a certain degree of risk aversion of the decision maker.

### Water level management and energy production at the Upper Atbara Dam

The Upper Atbara Dam Complex has a watershed area of about 100,000 $\hbox {km}^2$^46^. The location of the dam in the Atbara basin is shown in Fig. 1. The complex was built as a twin dam just upstream of the confluence of the Upper Atbara and Setit rivers with a power generation of up to 320 MW. The Full Supply Level (FSL) of the Upper Atbara Dam Complex is at 521 m asl, the Minimum Operation Level (MOL) or Sluicing Level is at 510 m asl.

A spreadsheet simulation model was applied based on the reservoir operation model that had been developed by the responsible dam planning company Tractebel Engineering GmbH to guide the reservoir impounding during and after the dam construction period for the Dam Implementation Unit (DIU) in Sudan, i.e. the stakeholder responsible for the erection of this scheme. The model considers the necessary components of a mass balance and power production, such as inflow, reservoir operation rules, an elevation-volume-area curve, evaporation from the reservoir surface, a tailwater rating curve, hydraulic losses, efficiencies for turbines, generator and transformer, and own electrical energy consumption.

The main purpose of the reservoir operation rule is maximizing the energy production while not leading to excessive reservoir sedimentation and in turn reducing reservoir storage. Thus, the reservoir water surface is lowered either down to sluicing level for sediment transport and removal (case of sluicing)^47^, or to a reservoir level that is able to absorb the peak inflows of the rainy season (no sluicing). Subsequent to the sluicing or water surface lowering operation, reservoir filling is initiated. Energy production usually maximizes during reservoir filling due to high inflows towards the end of the rainy season.

Inflow data provided by the DIU in Sudan and used in the simulation model of Tractebel Engineering GmbH were recorded on a daily basis at gauging stations Kubur/El Sofi and Wad El Heliew at the Upper Atbara river and at the Setit river, respectively. Both gauging stations were located upstream of the dam with a negligible intermediate catchment between their locations and the twin dam. These data were also used for the construction planning of the Upper Atbara Dam. For the calculations of drought- and standard-reservoir operations, the daily inflow of the year 2015 was taken as an example for a dry year. For a normal year, an average inflow of the period 1963-2016 was chosen. The latter ensures a reservoir management designed for average conditions and average energy production. To convert the produced energy into US$, a price of 0.2 US$ per kWh was assumed.

## Supplementary Information

Supplementary Information 1.

## Data Availability

Basin-averaged seasonal forecast and reanalysis data that support the findings of this study are available under https://radar.kit.edu/radar/en/dataset/XqHmFKADBYNNKqTi (doi: 10.35097/441). Regarding the reservoir operations at the Upper Atbara Dam, the supporting data are available from Tractebel Engineering GmbH but restrictions apply to the availability of these data, which were used under license for the current study, and so are not publicly available. Data are however available from the authors upon reasonable request and with permission of Tractebel Engineering GmbH. Data of the Multivariate ENSO Index Version 2 (MEI.v2) can be accessed via https://www.psl.noaa.gov/enso/mei/ (Supplementary Information 1).
