# Publisher Correction: Seasonal forecasts offer economic benefit for hydrological decision making in semi-arid regions

**DOI:** 10.1038/s41598-021-96668-y

**Published:** 2021-08-19

**Authors:** Tanja C. Portele, Christof Lorenz, Berhon Dibrani, Patrick Laux, Jan Bliefernicht, Harald Kunstmann

**Affiliations:** 1 Karlsruhe Institute of Technology (KIT), Campus Alpin, Institute of Meteorology and Climate Research - Atmospheric Environmental Research (IMK-IFU), Garmisch‑Partenkirchen, Germany; 2 Tractebel Engineering GmbH, Bad Vilbel, Germany; 3 University of Augsburg, Institute of Geography, Augsburg, Germany

Correction to: *Scientific Reports* https://doi.org/10.1038/s41598-021-89564-y, Published online 19 May 2021

The original version of this Article contained errors.

The citations to Figure 4 and Figure 5 were interchanged in the Results and Discussion sections.

In addition, in the legend of Figure 5,

“(see Equations 2a, 2b)”

now reads:

“(see Equation 2)”

Finally, in the Data Availability section,

“Basin-averaged seasonal forecast and reanalysis data that support the findings of this study are available under https://radar.kit.edu/radar/en/dataset/XqHmFKADBYNNKqTi?token=LOkISQJRuNCAMUMwUzjC (https://doi.org/10.35097/441). Repository KITopen of the Karlsruhe Institute of Technology with DOI identifiers. Repository KITopen of the Karlsruhe Institute of Technology with DOI identifiers. Regarding the reservoir operations at the Upper Atbara Dam, the supporting data are available from Tractebel Engineering GmbH but restrictions apply to the availability of these data, which were used under license for the current study, and so are not publicly available. Data are however available from the authors upon reasonable request and with permission of Tractebel Engineering GmbH. Data of the Multivariate ENSO Index Version 2 (MEI.v2) can be accessed via https://www.psl.noaa.gov/enso/mei/.”

now reads:

“Basin-averaged seasonal forecast and reanalysis data that support the findings of this study are available under https://radar.kit.edu/radar/en/dataset/XqHmFKADBYNNKqTi (https://doi.org/10.35097/441). Regarding the reservoir operations at the Upper Atbara Dam, the supporting data are available from Tractebel Engineering GmbH but restrictions apply to the availability of these data, which were used under license for the current study, and so are not publicly available. Data are however available from the authors upon reasonable request and with permission of Tractebel Engineering GmbH. Data of the Multivariate ENSO Index Version 2 (MEI.v2) can be accessed via https://www.psl.noaa.gov/enso/mei/ (Supplementary Information 1).”

The original Article has been corrected.

