# Supplementary material for: Seasonal forecasts offer economic benefit for hydrological decision making in semi-arid regions
Source: Sci Rep. 2021 May 19;11:10581. doi: 10.1038/s41598-021-89564-y (PMC8134578; doi:10.1038/s41598-021-89564-y)
Supplement: Supplementary file 1 — Supplementary Information 1. [file 41598_2021_89564_MOESM1_ESM.pdf]

Seasonal Forecasts offer Economic Benefit for Hydrological Decision Making in Semi-Arid Regions

Tanja C. Portele[1,*], Christof Lorenz[1], Berhon Dibrani[2], Patrick Laux[1,3,+], Jan Bliefernicht[3,+], and Harald Kunstmann[1,3,+]

[1]Karlsruhe Institute of Technology (KIT), Institute of Meteorology and Climate Research - Atmospheric Environmental Research (IMK-IFU), Garmisch-Partenkirchen, Germany
[2]Tractebel Engineering GmbH, Bad Vilbel, Germany
[3]University of Augsburg, Institute of Geography, Augsburg, Germany
[*]tanja.portele@kit.edu
[+]these authors contributed equally to this work

SUPPLEMENTARY INFORMATION

Supplementary Results

As stated in the main text, the Chira basin was excluded in the analysis of increasing occurrence of extreme events in the recent decades (Fig. 2), as it does not show significant trends there. Instead, for the Chira basin, elevated frequencies of warm and wet months are significantly correlated to El Niño (Multivariate ENSO Index, MEI, Version 2[1] > 1), and increased frequencies of cold months significantly to La Niña (MEI< -1) at $\alpha = 0.05$ (Supplementary Fig. S1; also see Supplementary Methods for MEIv2). Except for the Karun basin with significant correlation for the frequencies of $T <$Q20 and MEI> 1 (El Niño), no temperature or precipitation extreme event of the other study basins was significantly correlated with either El Niño or La Niña.

Supplementary Figure

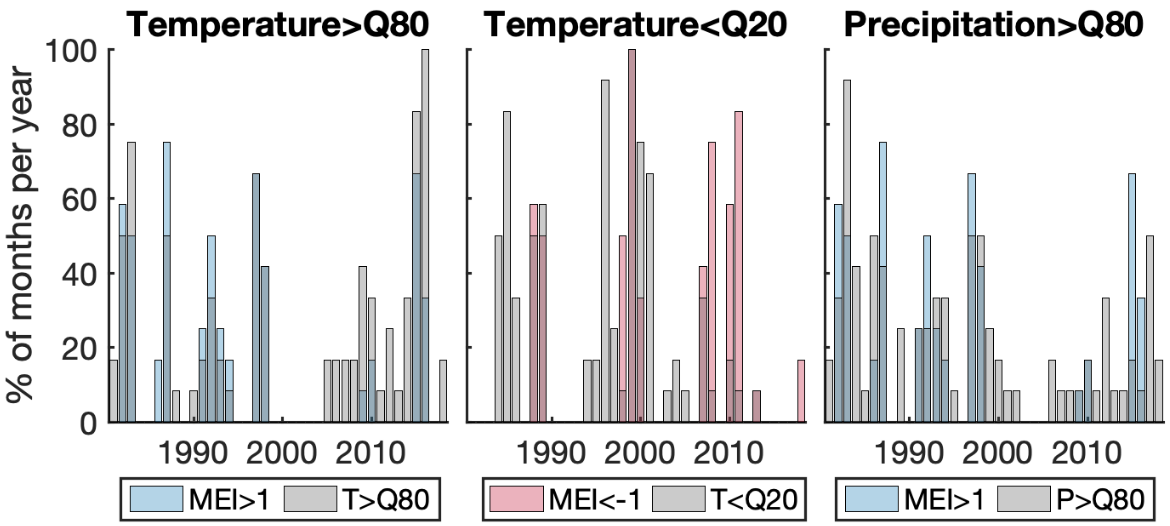

Figure S1. Relative frequency of climate variables for the Catamayo-Chira basin from 1981-2018. Grey bars show the relative frequency of ERA5 mean temperature $>$ Q80 (left) and $<$ Q20 (middle), and total precipitation $>$ Q80 (right). Blue and red bars depict the relative frequency of multivariate ENSO index MEIv2 > 1 (El Niño) and < -1 (La Niña), respectively.

Supplementary Methods

Drought indices

The Standardized Precipitation Index (SPI)[2] is calculated by the following steps:

1. Define the monthly precipitation dataset for an at least 30-year period, separately for the ensemble forecast and the reference.
2. Aggregate each dataset over the selected timescales, e.g., over $i = 1, 3, 4$ or 6 months, in a forecast - not in a retrospective - manner. This is done movingly in the sense that for each month new aggregated values are calculated from the current and the next $i-1$ months. That means for SPI6 of June, values from June to November are accumulated. For the forecasts, this implies using lead 0-5 from the forecast issued in June for each ensemble member.
3. Determine the cumulative probability distributions of the respective aggregated values for each start month including all available years for the reference, and all available years and ensemble members for the forecast. For not being dependent on the fit of parametric statistical distributions, we chose to use the empirical distribution for the calculation of SPI. Please note, the empirical cumulative probability distributions are separately calculated for reference and forecasts, instead of merging them, to allow the use of uncorrected, probably biased forecasts.
4. Estimate the inverse normal of the probability of aggregated values to determine the SPI value for the each data point, i.e., the derived deviation for a standard normally distributed probability density with zero mean.

With separate distributions for reference and forecasts, current values are only compared within the system's (reference or forecast) distribution. A chosen threshold of SPI< -1 thus defines a system's specific quantile value, representative of one negative standard deviation from the system's mean. Accordingly, standard bias correction approaches like linear scaling (correction of the mean) or quantile mapping (correction of absolute quantile values) at the same temporal (monthly) and spatial (basin-mean) resolutions have no effect.

For the Standardized Precipitation Evapotranspiration Index (SPEI), a simple water balance between precipitation (P) and potential evapotranspiration (PET) is calculated to derive the water deficit or surplus D. According to Vicente-Serrano et al., 2010[3], for the use of PET in the drought index, the method to calculate PET is not critical. Therefore, we followed their calculation of monthly PET (mm) with the simple approach by Thornthwaite 1948[4], requiring only data on monthly-mean temperature (T in °C):

$$\mathrm{PET} = 16K\left(\frac{10T}{I}\right)^{m} \tag{S1}$$

For the temperature-dependent heat index I, the coefficient m depending on I and the correction coefficient K depending on latitude and month, we refer to Vicente-Serrano et al., 2010[3]. The water deficit or surplus is then calculated for each year, month and lead time, separately for the forecasts and reference, as

$$D = P - \mathrm{PET}. \tag{S2}$$

Similarly to SPI, the derived values of D are then aggregated over different timescales and above steps 2-4 are applied.

Bootstrapping algorithm

As stated in the main text, the bootstrapping algorithm is applied to estimate the uncertainty of the hit rate H, false alarm rate F and the potential economic value PEV that may come along due to sampling errors of the considered extreme events. The applied algorithm for pairwise data is based on Bliefernicht et al., 2019[5] and Efron and Gong, 1983[6]. First, the bootstrap sample size m (here 1000) is defined. For each event ($T > Q80$, $P > Q80$, SPI< -1 and SPEI< -1), each target region and each probability threshold p_{th}, the following steps are applied to calculate the bootstrapped H, F and PEV:

1. Define the binary forecast-reference pairs of sample size n each. The reference time series $\mathbf{x} = (x_1, x_2, ..., x_n)$ becomes binary according to the event threshold x_{th} (e.g., $Q80$), and the event-based forecast probability $\mathbf{p} = (p_1, p_2, ..., p_n)$ is transformed to a binary array according to the current probability threshold p_{th}.
2. Resample the pairs of binary time series by randomly selecting with replacement (one sample pair can also be selected several times) n new binary forecast-reference pairs.
3. Calculate H, F and PEV for each new binary forecast-reference pair.
4. Repeat steps 2 and 3 m-times to obtain m bootstrapped versions of H, F and PEV.
5. To assess the robustness of forecast thresholds[7], calculate the confidence intervals for PEV based on the 10 % and 90 % quantiles of the m bootstrap samples of PEV.

Mann-Kendall trend significance test

The Mann-Kendall test is a non-parametric test for trend significance, that does not require any particular distribution of the tested timeseries[8]. The null hypothesis of the test is the absence of consistently increasing or decreasing trend in a timeseries $\mathbf{x} = (x_1, x_2, ..., x_n)$. The test analyzes differences in signs between earlier and later data points. The Mann-Kendall statistic S is defined as

$$S = \sum_{i=1}^{n-1} \sum_{j=i+1}^{n} \operatorname{sgn}(x_j - x_i), \tag{S3}$$

where n is the length of the timeseries and sgn denotes the sign function that allows values of $+1$, 0 and -1. For increasing or decreasing trends, the value of the Mann-Kendall statistic S should be highly positive or negative, respectively. To statistically test the trend, the probability associated with the Mann-Kendall statistic S is required. A normal distribution of the Mann-Kendall statistic S can be assumed for datasets with more than 10 sample points and including less equal values, i.e. ties. The normalized test statistic, the Z-value associated with S, is calculated as

$$Z = \begin{cases} \dfrac{(S-1)}{\sqrt{(VAR(S))}}, & \text{if S>0} \quad \text{(S4a)} \\ 0, & \text{if S=0} \quad \text{(S4b)} \\ \dfrac{S+1}{\sqrt{VAR(S)}}, & \text{if S<0,} \quad \text{(S4c)} \end{cases}$$

where the variance of the Mann-Kendall statistic $VAR(S)$ for a non-tied dataset is defined as

$$VAR(S) = \frac{1}{18} \cdot \{n(n-1)(2n+5)\}. \tag{S5}$$

Finally the probability (p-value) associated with the Z-value is calculated using the standard normal cumulative distribution function for a two-tailed test. Based on the chosen significance level α, typically 0.05, the null hypothesis is accepted or rejected. If the p-value of the test is less than α, the test rejects the null hypothesis, i.e., the test signifies the presence of trend in $\mathbf{x}$. Otherwise, if the p-value is greater than α, the null hypothesis of trend absence is accepted.

Multivariate ENSO Index Version 2 (MEIv2)

The phases of El Niño Southern Oscillation (ENSO) are described by the Multivariate El-Niño-Southern-Oscillation (ENSO) Index Version 2 (MEIv2)[1] that is based on the principal-component analysis of standardized anomalies of sea level pressure, sea surface temperature, 10-m zonal and meridional wind, and outgoing longwave radiation. For consistency with the monthly analysis, the two-month MEIv2 product (i. e., data for December-January, January-February, ... November-December) was linearly interpolated to monthly values. Data are available since 1979.

References

1. Zhang, T. *et al.* Towards Probabilistic Multivariate ENSO Monitoring. *Geophys. Res. Lett.* **46**, 10532–10540, DOI: 10.1029/2019GL083946 (2019).
2. McKee, T. B., Doesken, N. J. & Kleist, J. The relationship of drought frequency and duration to time scales. In *Eighth Conference on Applied Climatology*, 179–184 (American Meteorological Society, Anaheim, CA., 1993).
3. Vicente-Serrano, S. M., Beguería, S. & López-Moreno, J. I. A Multiscalar Drought Index Sensitive to Global Warming: The Standardized Precipitation Evapotranspiration Index. *J. Clim.* **23**, 1696–1718, DOI: 10.1175/2009JCLI2909.1 (2010).
4. Thornthwaite, C. W. An Approach toward a Rational Classification of Climate. *Geogr. Rev.* **38**, 55–94, DOI: 10.2307/210739 (1948).
5. Bliefernicht, J. *et al.* Quality and Value of Seasonal Precipitation Forecasts Issued by the West African Regional Climate Outlook Forum. *J. Appl. Meteorol. Clim.* **58**, 621–642, DOI: 10.1175/JAMC-D-18-0066.1 (2019).
6. Efron, B. & Gong, G. A Leisurely Look at the Bootstrap, the Jackknife, and Cross-Validation. *Amer. Stat.* **37**, 36–48, DOI: 10.1080/00031305.1983.10483087 (1983).
7. Lopez, A. *et al.* Bridging forecast verification and humanitarian decisions: A valuation approach for setting up action-oriented early warnings. *Wea. Clim. Extrem.* 100167, DOI: 10.1016/j.wace.2018.03.006 (2018).
8. Kendall, M. G. *Rank correlation methods* (Charles Griffin, London, 1975), 4th edn.
